# *Cistanche tubulosa* phenylethanoid glycosides suppressed adipogenesis in 3T3-L1 adipocytes and improved obesity and insulin resistance in high-fat diet induced obese mice

**DOI:** 10.1186/s12906-022-03743-6

**Published:** 2022-10-13

**Authors:** Dilinazi Abudujilile, Weilan Wang, Alimu Aimaier, Lili Chang, Yuliang Dong, Yiye Wang, Xu Fan, Yu Ma, Yongli Wang, Dilinigeer Ziyayiding, Yuan Ma, Jie Lv, Jinyao Li

**Affiliations:** 1 Xinjiang Key Laboratory of Biological Resources and Genetic Engineering, College of Life Science and Technology, Xinjiang University, Urumqi, 830017 China; 2 College of Resource and Environment Sciences, Xinjiang University, Urumqi, 830017 China

**Keywords:** *Cistanche tubulosa* phenylethanoid glycosides, Adipocyte, Obesity, Insulin resistance, Gut microbiota

## Abstract

**Background:**

*Cistanche tubulosa* is an editable and medicinal traditional Chinese herb and phenylethanoid glycosides are its major components, which have shown various beneficial effects such as anti-tumor, anti-oxidant and neuroprotective activities. However, the anti-obesity effect of *C. tubulosa* phenylethanoid glycosides (CTPG) and their regulatory effect on gut microbiota are still unclear. In the present study, we investigated its anti-obesity effect and regulatory effect on gut microbiota by 3T3-L1 cell model and obesity mouse model.

**Methods:**

3T3-L1 adipocytes were used to evaluate CTPG effects on adipogenesis and lipids accumulation. Insulin resistant 3T3-L1 cells were induced and used to measure CTPG effects on glucose consumption and insulin sensitivity. High-fat diet (HFD)-induced C57BL/6 obese mice were used to investigate CTPG effects on fat deposition, glucose and lipid metabolism, insulin resistance and intestinal microorganism.

**Results:**

In vitro data showed that CTPG significantly decreased the triglyceride (TG) and non-esterified fatty acid (NEFA) contents of the differentiated 3T3-L1 adipocytes in a concentration-dependent manner without cytotoxicity, and high concentration (100 µg/ml) of CTPG treatment dramatically suppressed the level of monocyte chemoattractant protein-1 (MCP-1) in 3T3-L1 mature adipocytes. Meanwhile, CTPG increased glucose consumption and decreased NEFA level in insulin resistant 3T3-L1 cells. We further found that CTPG protected mice from the development of obesity by inhibiting the expansion of adipose tissue and adipocyte hypertrophy, and improved hepatic steatosis by activating AMPKα to reduce hepatic fat accumulation. CTPG ameliorated HFD-induced hyperinsulinemia, hyperglycemia, inflammation and insulin resistance by activating IRS1/Akt/GLUT4 insulin signaling pathway in white adipose tissue. Moreover, gut microbiota structure and metabolic functions in HFD-induced obese mice was changed by CTPG, especially short chain fatty acids-producing bacteria including *Blautia, Roseburia, Butyrivibrio* and *Bacteriodes* were significantly increased by CTPG treatment.

**Conclusions:**

CTPG effectively suppressed adipogenesis and lipid accumulation in 3T3-L1 adipocytes and ameliorated HFD-induced obesity and insulin resistance through activating AMPKα and IRS1/AKT/GLUT4 signaling pathway and regulating the composition and metabolic functions of gut microbiota.

**Supplementary Information:**

The online version contains supplementary material available at 10.1186/s12906-022-03743-6.

## Background

Obesity is mainly caused by an imbalance of energy intake and consumption in white adipose tissue (WAT), and predisposes many metabolic disorders such as type 2 diabetes mellitus (T2DM), hyperlipidemia and cardiovascular diseases [1, 2]. Insulin resistance (IR) is a typical pathological feature and disease basis of obesity [3]. Many studies have found that adipose tissue is the initial site of IR generation [4–7]. In obesity, hyperplasia (cell number increase) and hypertrophy (cell size increase) of WAT is accompanied by increased levels of triglycerides and NEFA in plasma [8, 9], resulting in lipid deposition into plasma and tissues, such as liver and muscle, simultaneously triggering low-grade inflammation [10–13], reducing insulin sensitivity in adipose tissue, liver and skeletal muscle via insulin receptor substrate1 (IRS1)/AKT mediated insulin signaling [14–17], and ultimately leading to systemic insulin resistance and glucose and lipid metabolism disorders [18, 19]. Thus, inhibition of adipogenesis and lipid accumulation and improvement of adipose tissue function are extremely important in the prevention and treatment of insulin resistance in obesity.

In recent years, growing interest has focused on the modulation of gut microbiota as a therapeutic strategy in metabolic disorders [20, 21]. The increasing evidence shows that gut microbiota may play an important role in the pathogenesis of metabolic diseases, such as obesity, IR and T2DM [22–25]. Intestinal microorganisms are capable of producing large amounts of metabolites, some of which are absorbed directly into the systemic circulation [26]. Numerous studies have reported that obesity can alter the gut microbiota composition and metabolic function, shift the ratio of gut *Firmicutes* to *Bacteroides*, suppress some favorable bacteria, while stimulate the potentially harmful bacteria [27–30]. The abnormal alteration of gut microbiota, called dysbacteriosis, is associated with IR in obesity [31, 32]. Dysbacteriosis directly influences the host metabolic health including the physiological disorders by secondary metabolites including lipopolysaccharide (LPS) and short chain fatty acids (SCFAs) [33]. SCFAs are pivotal secondary metabolites of gut microbiota, which can inhibit inflammatory response and IR [34]. Consequently, the gut microbiota might be a novel target for the treatment of IR induced by obesity.

Traditional Chinese medicine (TCM) is widely used in the prevention and treatment of chronic metabolic diseases with multi-target and multi-active components. *Cistanche tubulosa* is one of the TCM with various pharmacological functions including anti-inflammation, anti-aging, neuroprotection, and other biological functions [35–38]. Phenylethanoid glycosides are the major bioactive components of *C. tubulosa* (CTPG) and contain effective components such as echinacoside and acteoside. CTPG also have various biological activity including anti-oxidation, anti-inflammation, neuroprotection and hepatocyte protection [39]. Our previous studies demonstrated﻿ that CT﻿PG had anti-tumor effect both in *vitro* and in *vivo* [40, 41]. In addition, it also has been reported that the water extract of *C. tubulosa* can suppress the elevated fasting blood glucose and postprandial blood glucose levels, improve IR and dyslipidemia in db/db mice [42]. Another study further demonstrated that the principal constituents of *C.tubulosa* including acylated phenylethanoid glycosides, echinacoside and acteoside inhibited the increased postprandial blood glucose levels and improved glucose tolerance in starch-loaded mice [43]. However, the anti-diabetic activity of CTPG has not been fully investigated.

In the present study, we evaluated the effects of CTPG on 3T3-L1 adipocytes differentiation, adipogenesis, glucose homeostasis and investigated its underlying effect and mechanism of CTPG on mice with high-fat diet-induced obesity, especially the regulation of gut microbiota.

## Methods

### Materials

CTPG (> 85% purity) was purchased from Shanghai Angbu Biotech Co., Ltd. (SGJG20170410, Shanghai, China) and dissolved by distilled water at the concentration of 100 mg/ml. The major compounds of CTPG were qualified and quantified by high performance liquid chromatography (HPLC) which contained 28% echinacoside, 9.9% acteoside [44]. The echinacoside and acteoside standards (Yuanye, Shanghai, China) were used to analyze the components of CTPG.

### Experimental design

Firstly, the effect of CTPG on adipocyte differentiation, lipid accumulation and inflammation in vitro was investigated using differentiated 3T3-L1 adipocytes model. Then, the effects of CTPG on insulin sensitivity, lipid metabolism and glucose homeostasis were investigated in IR-3T3-L1 adipocytes. Finally, we observed the effects of CTPG on the morphology and size of adipose tissue and hepatic steatosis of C57BL/6 obese mice induced by high-fat diet, and explored the regulatory effects and mechanism of CTPG on glucose and lipid metabolism, insulin resistance, inflammation and gut microbiota of obese mice.

### Cell culture and differentiation

The mouse 3T3-L1 cells were purchased from iCell Bioscience Inc. (Shanghai, China). Undifferentiated 3T3-L1 cells were cultured in DMEM medium containing 10% Newborn Calf Serum (NBS) for the around 70% confluent, then these cells (day 0) were treated with MDI contained 0.5 mM 3-isobutyl-1-methylxanthine (IBMX), 1 μM dexamethasone (DEX), 10 μg/ml insulin, and 10 μM rosiglitazone (RG) in DMEM medium with 10% fetal bovine serum (FBS), at 37 °C in a humidified atmosphere of 5% CO_2_. The IBMX and DEX were withdrawn after 2 days (day 2), then the insulin was removed on day 4. Thereafter, the cells were maintained in DMEM supplemented with 10% FBS, and the medium was changed every 2 days until day 8.

### Cell viability

In order to screen the safe doses of CTPG, MTT assay was used to determine the viability of 3T3-L1 preadipocytes. Briefly, cells were seeded in a 96-well cell culture plates at density of 5 × 10^3^ cells/well. Cells were treated with CTPG (25, 50, 75, 100 and 200 μg/ml) for 48 h. After washing, cells were incubated for 4 h with 5 mg/ml of MTT at 37℃. The formazan crystals were completely solubilized with dimethyl sulfoxide (DMSO), and absorbance was measured at 490 nm using microplate reader.

### Quantification of TG and MCP-1 content

3T3-L1 preadipocytes were treated with or without CTPG (25, 50, 75 and 100 μg/ml) from day 0 to day 8. On day 8, the cells were harvested and lysed followed by quantification of intracellular TG content (Nanjing Jiancheng, China), which was normalized to cell total protein levels using a BCA Protein Assay Kit (Thermo Fisher Scientific, USA). The cell supernatant was collected, and a NEFA kit (Nanjing Jiancheng, China) was used to detect the released NEFA. On day 6, the cell supernatant was collected, and the MCP-1 levels were measured by the mouse MCP-1 ELISA kit (Boster, China). All measurements were conducted according to the manufacturer's instructions.

### Oil Red O staining and quantification

For Oil Red O staining, 3T3-L1 adipocytes were washed with PBS and fixed with 4% formalin for 40 min, then washed with PBS for three times. After air drying, Oil Red O working solution was added to each well and placed at room temperature for 30 min, then the solution was removed. Cells were washed with distilled water for three times. The images of stained cells were photographed by inverted fluorescence microscopy (Nikon Eclipse Ti-E, Tokyo, Japan). For quantification, the Oil Red O in TG droplets was extracted with 100% isopropanol and the OD values were determined at 510 nm by a 96-well microplate reader (Bio-Rad Laboratories, Hercules, CA, USA).

### Induction of IR cell model and determination of glucose uptake

For the induction of IR cell model, on day8, the mature 3T3-L1 cells were treated with 1 μM DEX for 72 h. The IR-3T3-L1 cell model treated with or without CTPG (25, 50,75 and 100 μg/ml) for 48 h, then the glucose contents of supernatant were measured by Glucose oxidase–peroxidase kit (Shanghai Rongsheng Biotech, China), and the released NEFA was detected by NEFA kit (Nanjing Jiancheng, China) according to the manufacturer's instructions.

The glucose content in culture medium was measured with a glucose assay kit (Nanjing Jiancheng Biotechnology, China) according to the manufacturer’s instructions. The OD values at 505 nm was read in a microplate reader (Bio-Rad Laboratories, Hercules, CA, USA) and glucose consumption was calculated as the difference between initial glucose (IG) and extracellular glucose (EG) according to the equation: Glucose consumption = IG – EG.

### Establishment of mouse model

5-week-old male C57BL/6 mice were purchased from Animal Laboratory Center, Xinjiang Medical University (Urumqi, Xinjiang, China). All animals were housed in a standard temperature-controlled, light-cycled animal facility of Xinjiang University.

After 1 week of adaptive feeding, mice were fed either HFD (60% fat-derived calories, Research Diets, D12492) or NFD (11% fat-derived calories, Medicience, MD17121) beginning at 6 weeks of age. The detailed compositions of the two diets are presented in Table 1. The body weight and food intake of mice were measured weekly. After 14 weeks, the sera were collected after 6 h fasting. The NFD mice (*n* = 6) was used as a normal control. According to body weight and fasting plasma glucose, HFD mice were divided into three groups (6 mice/group), which were HFD group (model group without treatment), metformin (MET, positive control) group and CTPG treatment group.

**Table 1:** Composition of NFD and HFD

**Formula**	**HFD**		**NFD**	
	**gm%**	**Kcal%**	**gm%**	**Kcal%**
Protein	19.3	25	26.2	20
Carbohydrate	50.3	64	26.3	20
Fat	4	11	34.9	60

### Dosage information

The NFD mice were orally administered water (solvent of drug), and HFD mice were orally administered water, MET (250 mg/kg/day) or CTPG (300 mg/kg/day) with HFD every day for 6 weeks. Echinacoside and acteoside were the two main components of CTPG, accounting for 28% and 9.9% respectively [44]. Therefore, the dose of CTPG equivalent to 84 mg echinacoside/kg/day was chosen according to previous studies [42, 45].

### Serum biochemical analysis

Mice were fasted for 6 h after 6 weeks of drug administration and blood was collected to detect the blood glucose by a glucometer (Yuwell, China) and the levels of insulin by the mouse insulin ELISA kit (Elabscience, China). The levels of ALT, AST, TC and TG in mouse sera were determined by commercial kits (Nanjing Jiancheng, China). Sera GHbA1c levels were measured by the mouse GHbA1c ELISA kit (Hengyuan, China). All measurements were conducted according to the manufacturer's instructions.

### Assessment of insulin sensitivity in mice

Insulin sensitivity was also assessed by QUICKI using online-based calculator on the MedSci website (https://m.medsci.cn/scale/show.do?id=be21111057), and HOMA2 index using calculator from the Diabetes Trials Unit of the University of Oxford website (http://www.dtu.ox.ac.uk/homacalculator/download.php) providing values for HOMA2-IR, HOMA2-%B, and HOMA2-%S. QUICKI and HOMA are the most widely used indices for assessing insulin sensitivity based on fasting glucose and insulin measures, which are calculated according to the following formulas.

QUICKI = 1/(log (Fasting Insulin [mg/dL]) + log(Fasting Glucose[microU/mL])).

HOMA = (Fasting glucose [mmol/L] x Fasting insulin [microU/mL])/22.5

### OGTT and ITT

For OGTT, mice were fasted for 6 h after 5 weeks of drug administration, then orally administered with 2 g/kg of glucose. Blood glucose concentrations were measured at 0, 15, 30, 60, and 120 min after glucose treatment. The curves were made and AUC was calculated. For ITT, mice were fasted for 4 h after 6 weeks of drug administration, and then intraperitoneally injected with 0.75 U/kg insulin. Blood glucose levels were measured at 0, 15, 30, 60, and 120 min after insulin injection. The curves were made and the AUC was calculated.

### Quantification of lipid content in liver

After 6 weeks of administration, the mice liver was isolated. For liver lipid content determination, homogenize tissue at the ratio of the weight of the tissue (g): the volume of PBS (ml) = 1:9, then the tissue homogenate was centrifuged with 5,000 rpm for 10 min, and the supernatant was collected. Liver TG and TC content was assessed by TG kit (Nanjing Jiancheng, China) and TC kit (Nanjing Jiancheng, China), which was normalized to cell total protein levels using a BCA Protein Assay Kit (Thermo Fisher Scientific, USA).

### Histological analysis of liver and adipose tissue

Adipose tissues and livers were collected at the end of this study and fixed in 10% formaldehyde solution for 48 h, followed by embedding in paraffin and staining 5 μm-thick sections with hematoxylin and eosin (H&E). The cell sizes of epididymal WAT (eWAT), inguinal WAT (iWAT), and perirenal WAT (pWAT) were measured in H&E stained sections of three individual samples in each group. The sections were imaged by inverted fluorescence microscopy and quantified with Image J.

### Quantification of MCP-1 content in eWAT

After 6 weeks of administration, eWAT of the mice was isolated. In order to determine the MCP-1 content of eWAT, 1 ml PBS was added per 100 mg frozen eWAT tissue, and samples were centrifuged with 5,000 rpm for 10 min, and the supernatant was collected. eWAT MCP-1 content was assessed by the mouse MCP-1 ELISA kit (Boster, China).

### Quantitative RT-PCR (qRT-PCR)

The mRNA levels of nuclear receptor CCAAT enhancer-binding protein alpha (C/EBPα), acetyl-CoA carboxylase (ACC), fatty acid synthase (FASN), preadipocyte factor-1 (Pref-1) and hormone sensitive lipase (HSL) were detected by qRT-PCR. 3T3-L1 cells were treated with 100 μg/ml CTPG for 72 h and the total RNA was extracted by Cell total RNA isolation Kit (Foregene, China) according to the manufacturer’s protocol. Reverse transcription and quantitative PCR were performed according to the instructions of RT EasyTM II kit (Foregene, China) and RT-qPCR EasyTM (One Step)-SYBR Green I kit (Foregene, China), respectively. The gene-specific primers were shown in Table S1.

### Western blot analysis

At the end of this study, proteins were isolated from liver and eWAT using RIPA lysis buffer (Beyotime biotechnology, China) and protein concentrations were measured by BCA Kit (Thermo Fisher Scientific, USA). Equal amount of protein in each sample was separated by 10% SDS-PAGE and transferred to PVDF membranes (Biosharp, China). After blocked with 5% nonfat milk, membranes were incubated with corresponding primary antibodies and secondary antibodies conjugated to horseradish peroxidase, respectively. After washing with PBS contained 0.05% Tween 20, the proteins were detected by EasySee Western Kit (Transgene, Beijing), and the blots were analyzed by ImageJ [46].

### Gut microbiota analysis

About 100 mg cecum content from different group mice were used for DNA extraction using a cetyltrimethylammonium bromide (CTAB) protocol [46]. The DNA concentration was measured using Qubit® dsDNA Assay Kit by Qubit® 2.0 Flurometer (Life Technologies, CA, USA). The DNA samples were fragmented to an average size of about 350 bp using Covaris M220 (Gene Company Limited, China), then DNA fragments were end-polished, A-tailed, and ligated with the full-length adaptor for Illumina sequencing with further PCR amplification using NEBNext® Ultra™ DNA Library Prep kit (NEB, USA). At last, PCR products were purified and quantified for sequencing. Paired-end sequencing was performed on Illumina HiSeq2500 platform (Illumina Inc., San Diego, CA, USA) at Novogene Co., Ltd. (Beijing, China). Adapter sequence and low quality reads (length < 50 bp or with a quality value < 20 or having N bases) were removed by trimmomatic (v0.39) [47]. Reads were aligned to the Mouse Genome Database by bowtie2 (v2.3.4.3) software [48] to filter the reads that are of host origin, and then the remain reads were aligned to the download NCBI taxonomic information database using Kraken2 (v2.0.8-beta) [49] for taxonomic annotations. Clean reads were assembled using MEGAHIT (v1.1.3) [50] and contigs with the length over 300 bp were selected as the final assembling result, and then the contigs were used for further gene prediction and annotation by Prokka (v1.13.3) [51]. All predicted genes with the criteria of identity > 95% and overlap > 90% were clustered using CD-HIT (v4.8.1) [52], the longest sequences from each cluster were selected as representative sequences to construct non-redundant gene catalog. Reads after quality control were mapped to the representative sequences using Salmon (v0.14.0) [53] and gene abundance in each sample were evaluated.

The amino acid sequences of representative sequences were made on the basis of KofamKOALA [54] alignment against the KEGG database, and were annotated to the CAZy Database [55] using Diamond (v0.8.22). The microbial and functional diversity of three groups were determined and displayed using R packages.

### Predicted metabolic functions of gut microbiota in mice

The predicted orthologues based on KEGG database (https://www.genome.jp/kegg/) were summarized, and differential abundances by groups were determined and displayed using R packages.

### Statistical analysis

All data were expressed as the mean ± standard deviation (S.D.). The variance distribution was equaled using Bartlett’s test. Statistical analyses were performed using a one-way analysis of variance (ANOVA) conditions to the control followed by Dunnett's multiple comparisons test, with a 5% significance level. *p* < 0.05 was considered to be statistically significant.

## Results

### CTPG inhibited the lipid accumulation and increased glucose consumption in adipocytes

**Fig. 1:**
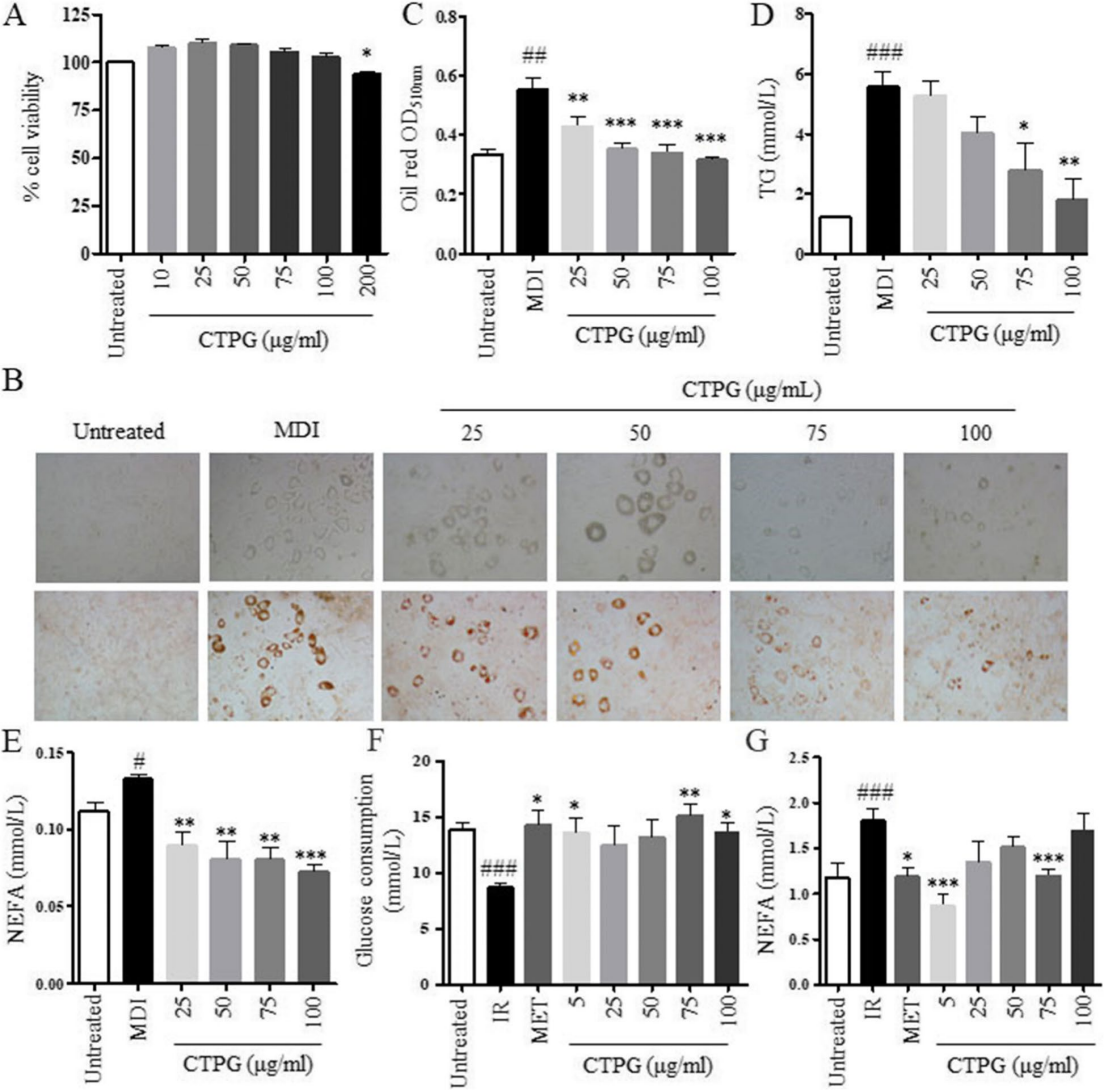
Effect of CTPG on lipid accumulation and glucose consumption in 3T3-L1 adipocytes. **A** Viability of 3T3-L1 cells treated with different concentrations of CTPG for 48 h. **B** The differentiation of 3T3-L1 preadipocytes in the presence or absence of CTPG for 8 days. Lipid droplets were stained using Oil Red O and observed using inverted fluorescence microscopy (× 200). **C** Quantitation of Oil Red O staining. **D** The concentrations of TG in 3T3-L1 adipocytes on day 8. **E** The concentrations of NEFA in the supernatant of 3T3-L1 adipocyte culture on day 8. **F** Glucose consumption in IR-3T3-L1 model after CTPG treatment for 48 h. **G** NEFA concentration in the supernatant of IR-3T3-L1 cell culture after CTPG treatment for 48 h. Data are expressed as mean ± SD. ^#^*p* < 0.05, ^##^*p* < 0.01 and ^###^*p* < 0.001, MDI or IR group versus Untreated group. ^*^p < 0.05, ^**^p < 0.01 and ^***^p < 0.001, CTPG, MET groups versus MDI or IR group

Cell viability was first examined by MTT assay to determine the optimum concentration of CTPG for further experiments. As shown in Fig. 1A, there was no significant change in 3T3-L1 cells viability at 25, 50, 75 and 100 μg/ml CTPG, as compared to the untreated group. The viability of 3T3-L1 cells was significantly decreased by 200 μg/ml CTPG. Therefore, the safe doses of CTPG including 25–100 μg/ml were used in further investigations.

The anti-adipogenic effects caused by CTPG on preadipocyte differentiation into adipocytes were investigated. 3T3-L1 preadipocytes were cultured in adipocyte differentiation media for 8 days in the presence or absence of CTPG. Then, we undertook Oil Red O staining to assess CTPG effects on adịpogenic differentiation at day 8 after the induction of adipocyte differentiation. The quantification of Oil Red O staining demonstrated that showed that 25 to 100 μg/ml of CTPG significantly inhibited lipid droplet accumulations versus that of differentiation mixture (MDI) induced model group in a dose-dependent manner (Fig. 1B&C). The contents of triglyceride (TG) and NEFA were also dose-dependently reduced by CTPG treatment (Fig. 1D&E).The effect of CTPG on IR-3T3-L1 cells was further detected. We observed that CTPG significantly increased glucose consumption (Fig. 1F) and decreased NEFA levels (Fig. 1G).

Next, we analyzed the effect of CTPG on adipogenesis of 3T3-L1 adipocytes by qRT-PCR. CTPG treatment significantly decreased the expression of adipogenic gene (C/EBPα) and lipogenic genes (ACC and FASN), while increased the expression of preadipocytic gene (Pref-1) and lipolysis gene (HSL) in 3T3-L1 cells (Fig. S1). These results indicated that CTPG suppressed 3T3-L1 adipocyte differentiation, decreased adipogenesis and intracellular lipid accumulation, and ameliorated IR in *vitro*.

### Establishment of HFD-induced IR mouse model

**Fig. 2:**
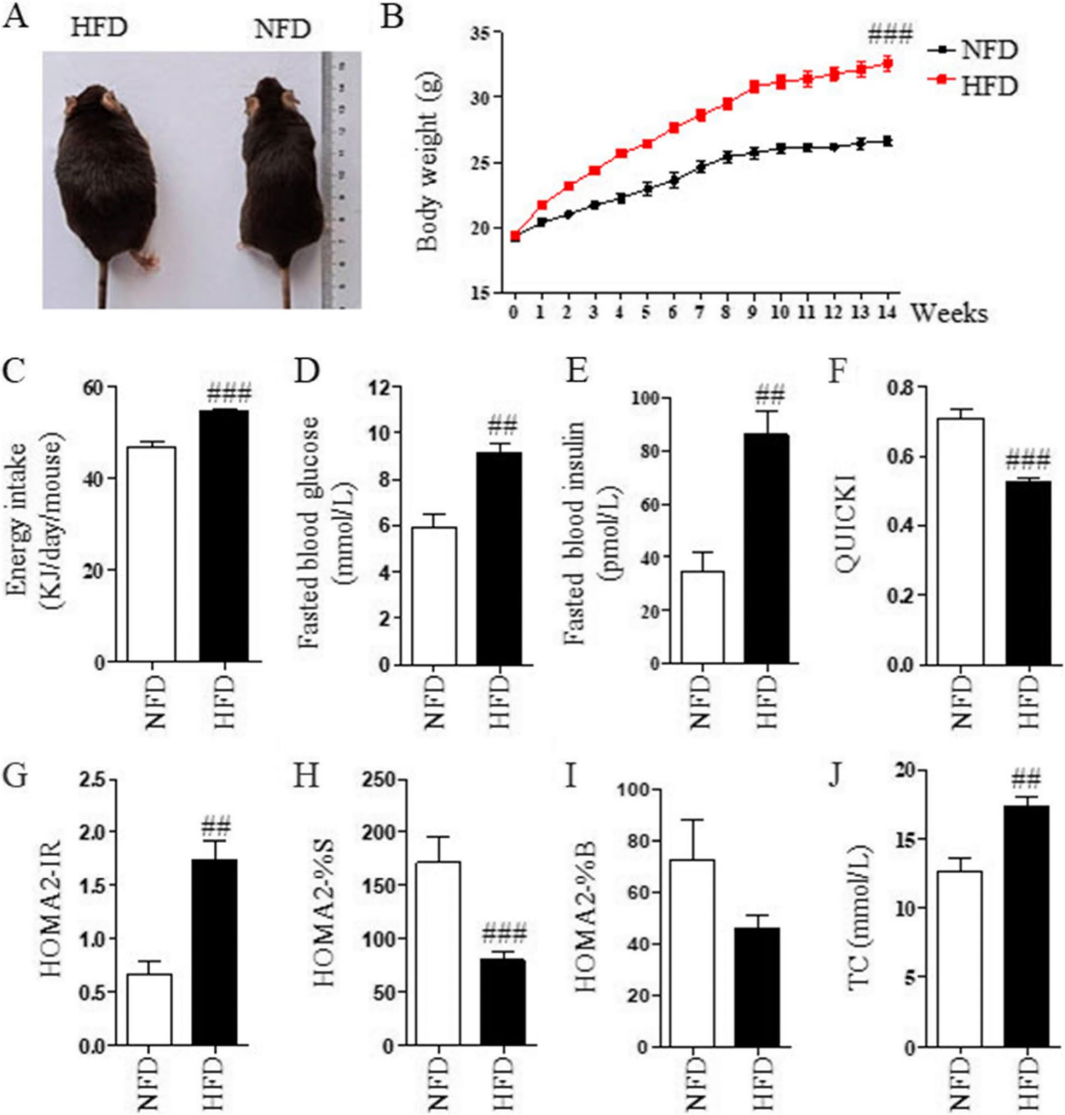
Characteristics of HFD-induced obese mice. C57BL/6 mice were fed with HFD for 14 weeks. Mice fed with NFD were used as control. **A** The picture of mice fed with HFD and NFD. **B** The body weight of mice fed with NFD (*n* = 7) and HFD (*n* = 43). **C** The energy intake. **D** The fasted blood glucose levels. **E** The fasted blood insulin levels. **F** QUICKI index values. **G** HOMA2 index values: insulin resistance (HOMA2-IR). **H** Insulin sensitivity (HOMA2-%S). (I) β-cell function (HOMA2-%B). **J** The levels of TC in serum. Data are expressed as mean ± SD. ^##^*p* < 0.01 and ^###^*p* < 0.001, compared with NFD group

To investigate the effect of CTPG on fat deposition and IR in vivo, the obese mouse model by feeding mice a high-fat diet was established. As shown in Fig. 2A, obese mice were successfully induced by high-fat diet (HFD) compared with normal-fat diet (NFD). The body weight (Fig. 2B) and energy intake (Fig. 2C) were monitored in both NFD and HFD groups. The body weight and energy intake in HFD group were significantly higher than that in NFD group. As expected, hyperglycemia and hyperinsulinemia were developed in the HFD mice, characterized by the elevated levels of fasting blood glucose and fasting blood insulin (Fig. 2D&E). Based on these measurements, the IR index was calculated by quantitative insulin sensitivity check index (QUICKI) and homeostasis model assessment-2 (HOMA2). Compared to NFD mice, HFD mice exhibited lower QUICKI and higher HOMA2 index-IR (HOMA2-IR) indexes (Fig. 2F&G). Similarly, the significantly impaired insulin sensitivity in HFD mice was observed based on HOMA2 index-insulin sensitivity (HOMA2-%S) values (Fig. 2H). As shown in Fig. 2I, there was a tendency for impaired β-cell function in HFD mice based on the HOMA2 index-β-cell function (HOMA2-%B). In addition, TC levels were significantly increased in HFD mice (Fig. 2J). Taken together, compared with NFD mice, HFD mice developed IR with lipid disorder.

### CTPG inhibited white adipose tissue expansion and fat accumulation in HFD-induced obese mice

**Fig. 3:**
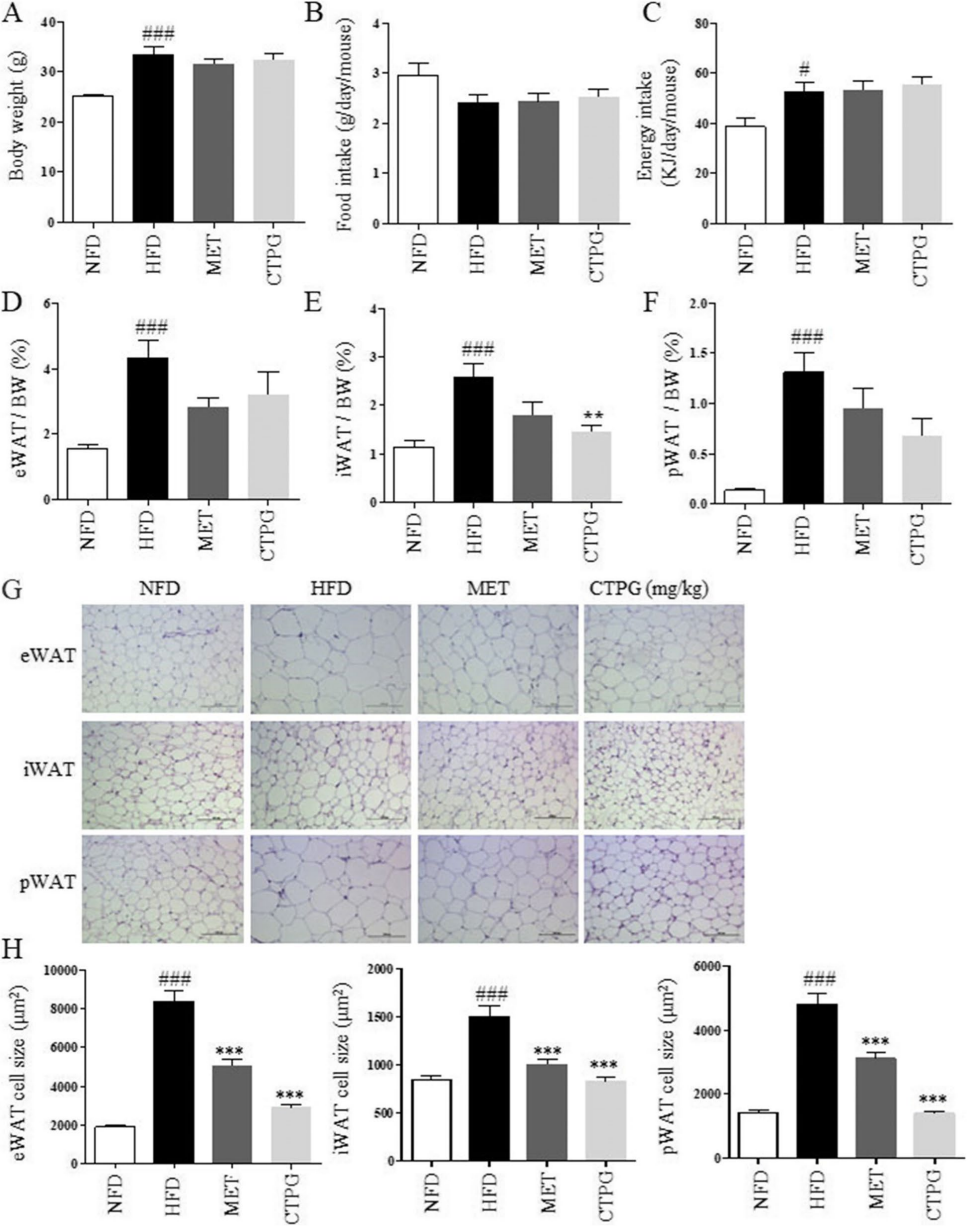
CTPG supplementation reduced fat accumulation and adipocyte size in HFD mice. HFD mice were treated with CTPG for 6 weeks and mouse body weight (**A**), food intake (**B**) and energy intake (**C**) were measured. The indexes of eWAT (**D**), iWAT (**E**) and pWAT (**F**) were calculated. **G** The sections of WAT were made and stained with H&E (× 200). **H** The sizes of adipocytes were evaluated by ImageJ. Data are expressed as mean ± SD. ^#^*p* < 0.05, ^##^*p* < 0.01 and ^###^*p* < 0.001, HFD group versus NFD group. ^*^p < 0.05, ^**^p < 0.01 and ^***^p < 0.001, CTPG and MET groups versus HFD group

HFD-induced obese mice were fed with HFD and orally treated with CTPG every day for 6 weeks. However, there was no difference in body weight (Fig. 3A), food intake (Fig. 3B) or energy intake (Fig. 3C) between the CTPG group (CTPG) and HFD group (HFD). At the end of the experiment, CTPG decreased the weight of eWAT (Fig. 3D), inguinal WAT (iWAT) (Fig. 3E), and perirenal WAT (pWAT) (Fig. 3F) compared with HFD group. Consistently, H&E staining showed that compared with the HFD group, eWAT, iWAT and pWAT adipocytes were smaller in the CTPG group, with a significantly decreased average cell area (Fig. 3G and H). These results suggested that CTPG effectively ameliorated HFD-induced obesity through decreasing the expansion of adipose tissues and adipocyte hypertrophy.

### CTPG reduced hepatic steatosis in HFD-induced obese mice

**Fig. 4:**
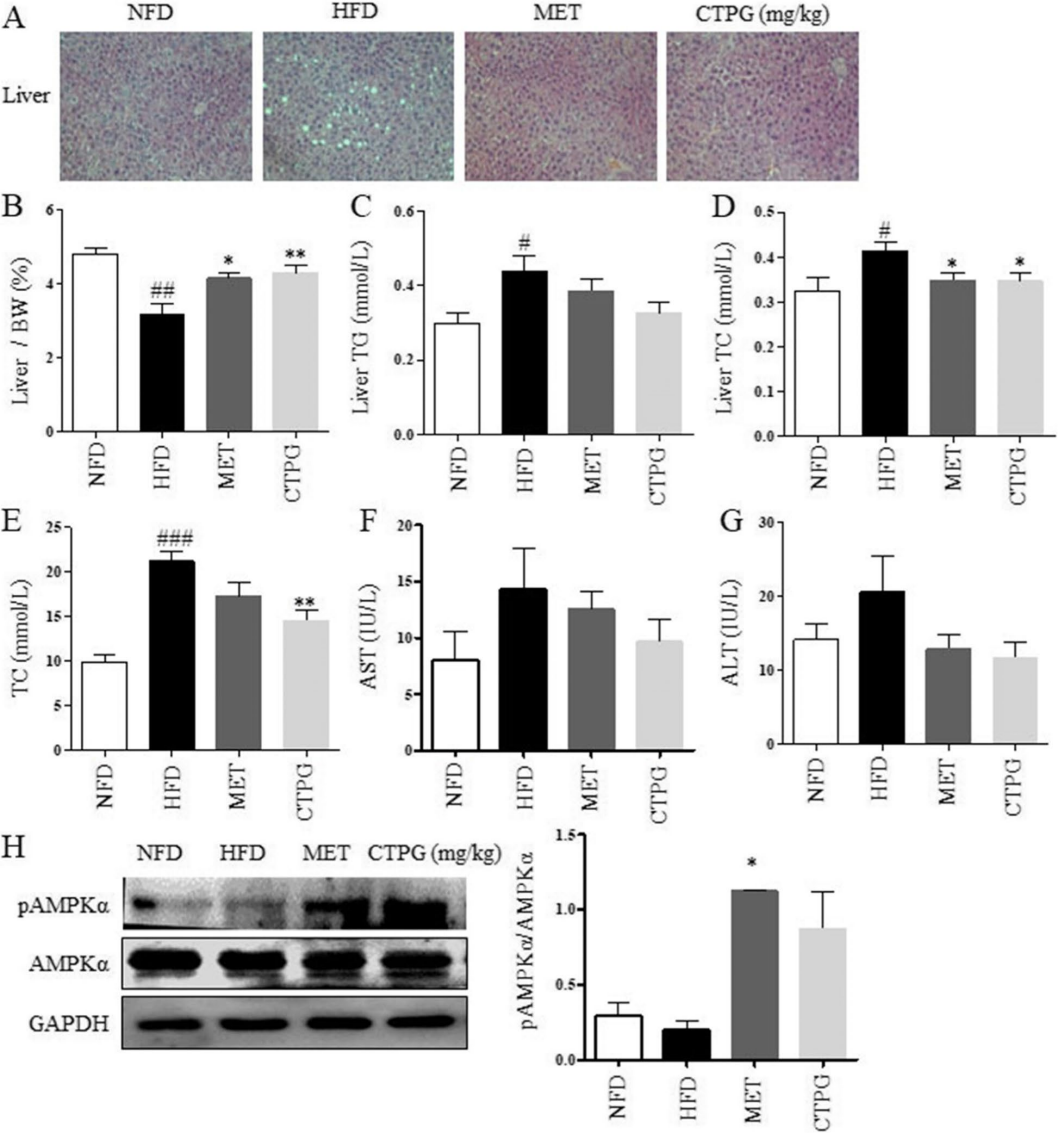
CTPG supplementation ameliorated hepatic steatosis and lipid profile in HFD mice. At the end of this study, mice were sacrificed to collect livers. **A** Liver sections were stained with H&E (× 200). **B** Liver indexes. The levels of TG (**C**) and TC (**D**) in livers were detected. The levels of TC (**E**), AST (**F**) and ALT (**G**) in serum were measured. **H** Total protein was isolated from livers and AMPKα protein expression and phosphorylation of AMPKα were detected by Western blot. Data are expressed as mean ± SD. ^#^*p* < 0.05, ^##^*p* < 0.01 and ^###^*p* < 0.001, HFD group versus NFD group. ^*^p < 0.05, ^**^p < 0.01 and ^***^p < 0.001, CTPG and MET groups versus HFD group

As an important metabolic center of the body, the liver is the main site for lipid production and accumulation. The anti-hepatic steatosis activity of CTPG in HFD-induced obese mice was examined. H&E staining showed that hepatic steatosis occurred in liver tissue of HFD mice, and there were obvious lipid droplets in hepatocytes, while CTPG administration could significantly improve the appearance of hepatic steatosis (Fig. 4A). Additionally, the liver index of the mice was decreased by HFD, which was also significantly increased in mice treated with CTPG (Fig. 4B). The TG and total cholesterol (TC) contents of liver in mice were by CTPG administration (Fig. 4C&D). Consistently, the levels of TC in sera were significantly reduced in CTPG group (Fig. 4E). Moreover, CTPG reduced the levels of aspartate aminotransferase (AST) and alanine aminotransferase (ALT) in sera compared with HFD mice, which alleviated HFD-induced liver damage (Fig. 4F&G). Thus, these results indicated that CTPG treatment improved the hepatic steatosis induced by HFD.

We assessed whether CTPG displays a key role on hepatic lipid metabolism and observed that the AMPKα phosphorylation in the liver, which was increased by CTPG (Fig. 4H).

### CTPG treatment ameliorates IR in HFD-induced obese mice

**Fig. 5:**
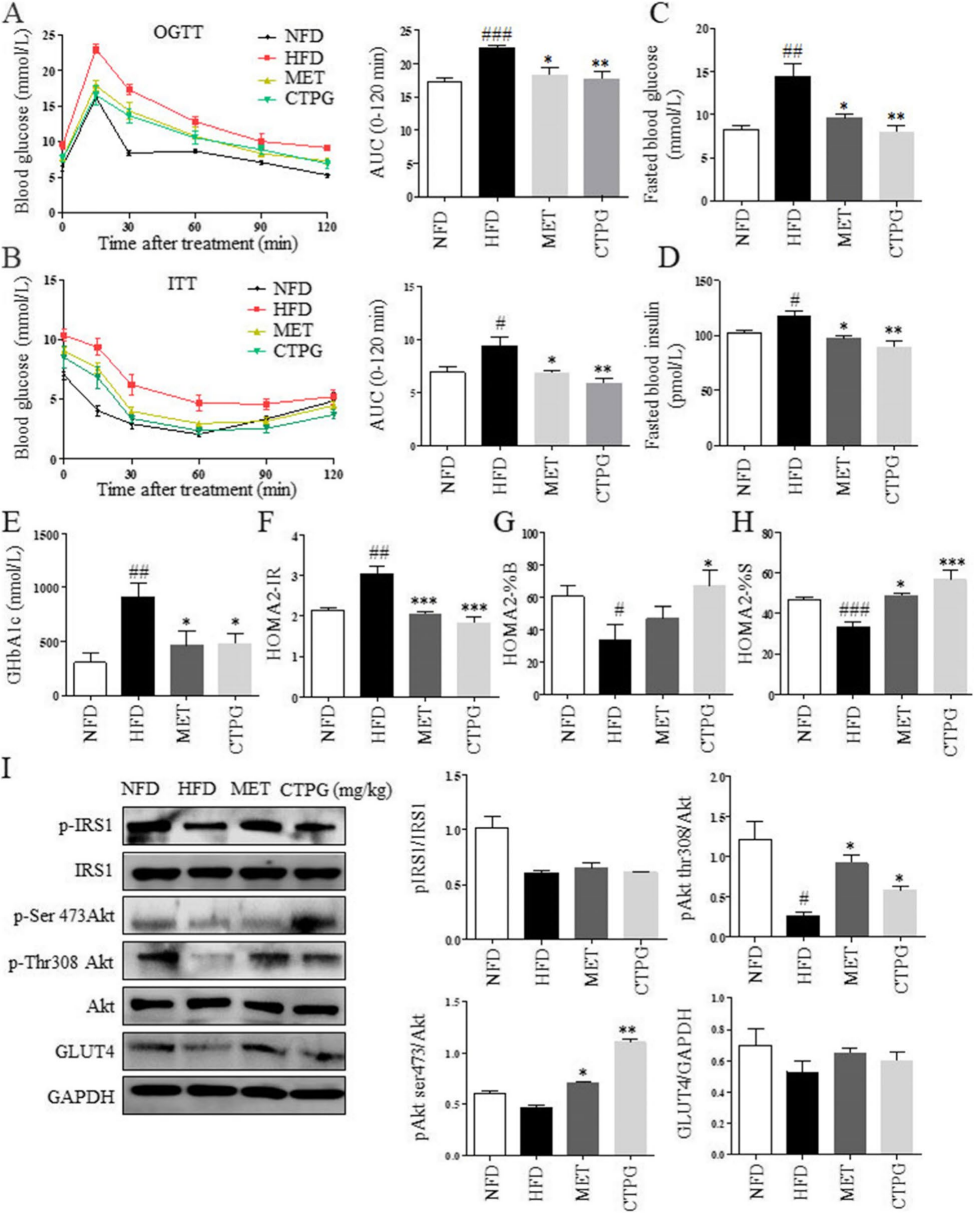
CTPG supplementation improved HFD-induced IR in mice. **A** OGTT. Blood glucose concentrations were measured at different time points to make curves and calculate AUC. **B** ITT. Blood glucose levels were measured at different time points to make curves and calculate AUC. Mice were fasted for 6 h after 6 weeks of CTPG supplementation and blood was collected to detect the levels of fasted blood glucose (**C**), fasted blood insulin (**D**) and serum GHbA1c (**E**). HOMA2-IR (**F**), β-cell function (HOMA2-%B) (**G**) and HOMA2-%S (**H**) were calculated. **I** Total protein was isolated from eWAT to detect protein expression and their phosphorylation in insulin signaling pathway by western blot. Data are expressed as mean ± SD. ^#^*p* < 0.05, ^##^*p* < 0.01 and ^###^*p* < 0.001, HFD group versus NFD group. ^*^p < 0.05, ^**^p < 0.01 and ^***^p < 0.001, CTPG and MET groups versus HFD group

The effect of CTPG on glucose tolerance in obese mice was measured with oral glucose tolerance test (OGTT) after 5 weeks of CTPG administration. As shown in Fig. 5A, the blood glucose level in HFD mice was rapidly increased up to 15 min after glucose administration, whereas that level in CTPG treated mice exhibited a moderate increase. The area under the curve (AUC) was lower in CTPG treated mice than HFD mice (*p* < 0.05). Then we investigated the effect of CTPG on IR by measuring insulin tolerance tests (ITT) and HOMA-IR index. The ITT was further tested after 6 weeks of CTPG treatment, the ITT curve increased moderately and the AUC index was smaller in CTPG and MET group (Fig. 5B). The levels of fasting serum glucose, insulin and glycated hemoglobin A1c (GHbA1c) were significantly decreased by CTPG treatment compared with the HFD mice (Fig. 5C-E). Consistently, the values of HOMA2-IR were significantly decreased by CTPG treatment (Fig. 5F), while the values of HOMA2-%B and HOMA2-%S were increased by CTPG treatment (Fig. 5G&H). These data suggested that CTPG restored glucose tolerance and enhanced insulin sensitivity in mice.

The effect of insulin on glucose is regulated by IRS1/Akt signaling pathway and the abnormality of the pathway can lead to the diminishment and impairment of insulin signaling [17, 19], which eventually result in disorders of glucose metabolism. The effect of CTPG on the expression of proteins related to IRS1/Akt/GLUT4 insulin signaling pathway was investigated. The results showed that the protein expression of GLUT4 was increased and phosphorylation of IRS1 and Akt in eWAT of HFD mice was increased by CTPG treatment (Fig. 5I). These data suggested that IRS1/Akt/GLUT4 insulin signaling was enhanced in adipose tissue of CTPG-treated HFD mice.

### CTPG reduced monocyte chemoattractant protein-1 (MCP-1) levels in vitro and in vivo

**Fig. 6:**
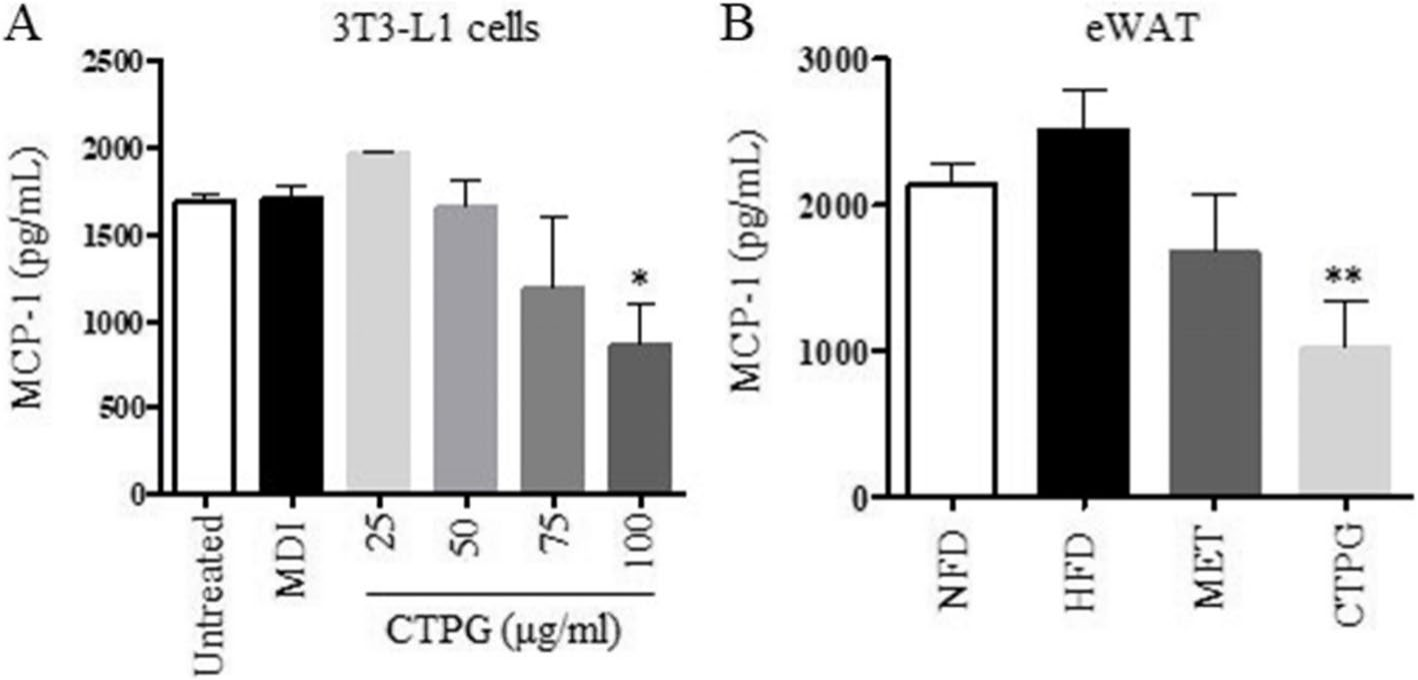
CTPG inhibited MCP-1 levels in vitro and in vivo. **A** The levels of MCP-1 were reduced by CTPG treatment in 3T3-L1 adipocytes. **B** The levels of MCP-1 were decreased in eWAT. Data are expressed as mean ± SD. ^##^*p* < 0.01 compared with NFD group. ^*^p < 0.05 compared with HFD group

WAT has emerged as a major endocrine organ, producing a variety of adipokines including MCP-1, which affect lipid metabolism and glucose homeostasis [11, 12]. The anti-inflammation effects of CTPG on adipocyte were examined by measurement of MCP-1 levels in vitro and in vivo. The levels of MCP-1 were dose-dependently reduced by CTPG treatment in 3T3-L1 adipocytes (Fig. 6A), and also decreased by CTPG in eWAT (Fig. 6B). These results indicated that CTPG inhibited MCP-1 levels in vitro and in vivo.

### CTPG regulated HFD-induced gut dysbiosis by modulated gut microbiota composition and structure

**Fig. 7:**
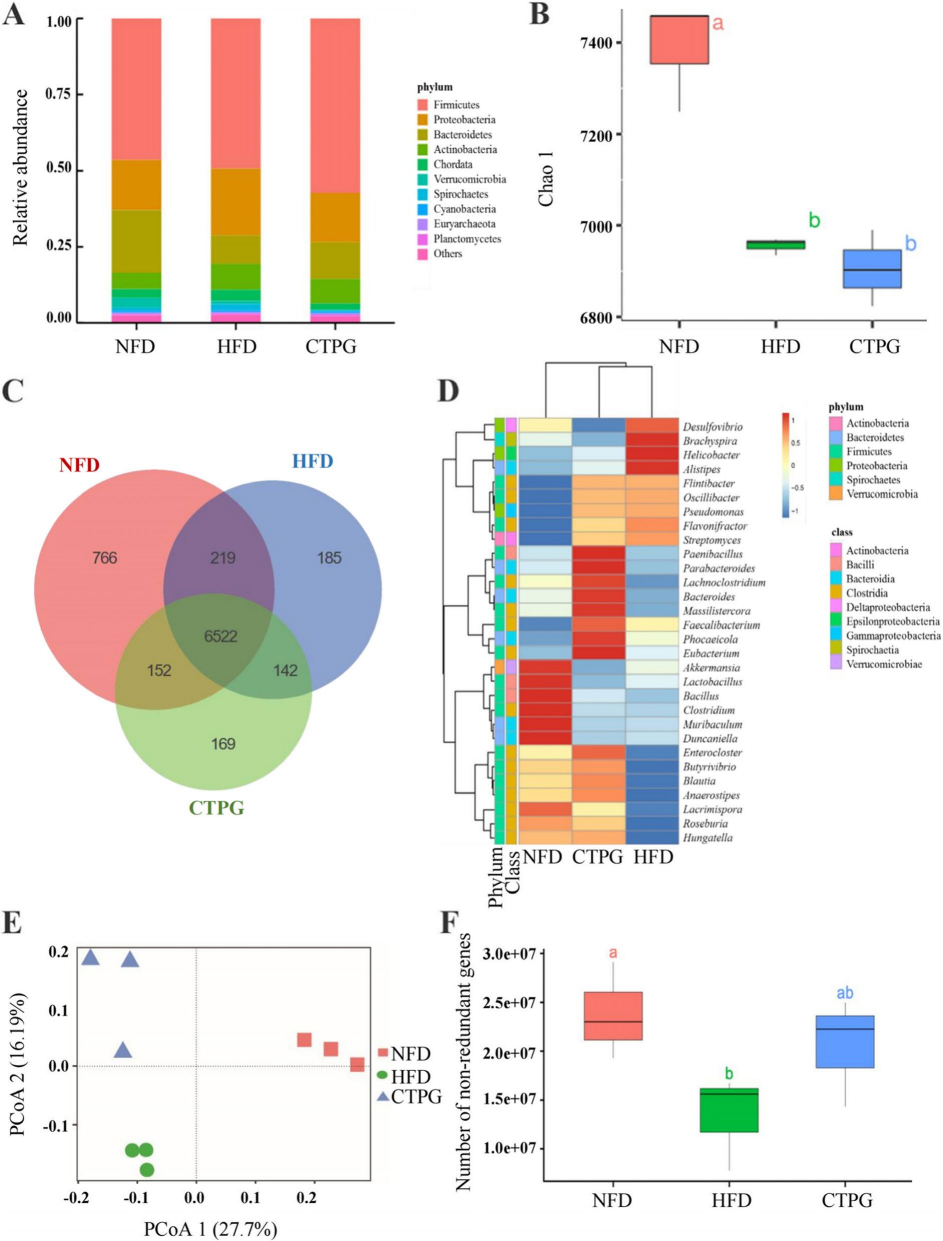
CTPG ameliorated HFD-induced gut dysbiosis. Cecum samples of NFD, HFD and CTPG groups (*n* = 3) were collected and analyzed by metagenomics. **A** The relative abundance of the top 10 phyla. **B** Chao1 index. **C** Venn diagram with numbers of species in gut microbiota and numbers of overlap species among the three groups. **D** Heatmap of top 30 genera. **E** PCoA analysis. **F** Gene numbers of gut microflora. Data are expressed as mean ± SD. Different lowercase letters indicate significant differences among groups (*p* < 0.05)

Gut microbiota changes are associated with obesity and metabolic syndrome. To analyze the structural and compositional changes of gut microbiota in NFD mice and HFD mice supplementing with or without 300 mg/kg CTPG for 6 weeks, cecum contents were collected and metagenomics sequencing was used to analyze microbial diversity. Taxonomic profiling of top 10 most abundance phylum indicated that HFD obviously increased the relative abundance of *Proteobacteria* and *Actinobacteria* phyla. Interestingly, *Proteobacteria* phylum was greatly reduced in mice gut after supplementation with CTPG (Fig. 7A). Compared with NFD group, Chao1 index was reduced significantly in the HFD group, indicating HFD significantly reduced microflora diversity in mice gut (Fig. 7B). The CTPG supplementation did not increase the total number of microbial species compared with HFD group but the composition of microbial species was altered (Fig. 7B&C), which were further confirmed by the analysis at the genus level. CTPG supplementation significantly increased the abundances of *Enterocloster*, *Butyrivibrio*, *Blautia*, *Anaerostipes*, *Lacrimispora*, *Roseburia* and *Hungatella*, which were similar with NFD groups. Moreover, CTPG supplementation also significantly increased the abundances of *Paenibacillus*, *Parabacteroides*, *Lachnoclostridium*, *Bacteroides*, *Massilistercora*, *Phocaeicola* and *Eubacterium* (Fig. 7D). Consistently, the data of principal coordinates analysis (PCoA) based on Bray–Curtis distance showed clear separation among NFD, HFD, and CTPG groups (Fig. 7E), suggesting that CTPG changed the gut microbial structure of HFD group. Additionally, the number of non-redundant genes was increased significantly by CTPG supplementation compared with HFD group (Fig. 7F). These results indicated that CTPG supplementation altered the structure and composition of gut microbiota in HFD mice, which might be associated with the improved metabolic disorders.

### Predicted metabolic functions of gut microbiota in mice

**Fig. 8:**
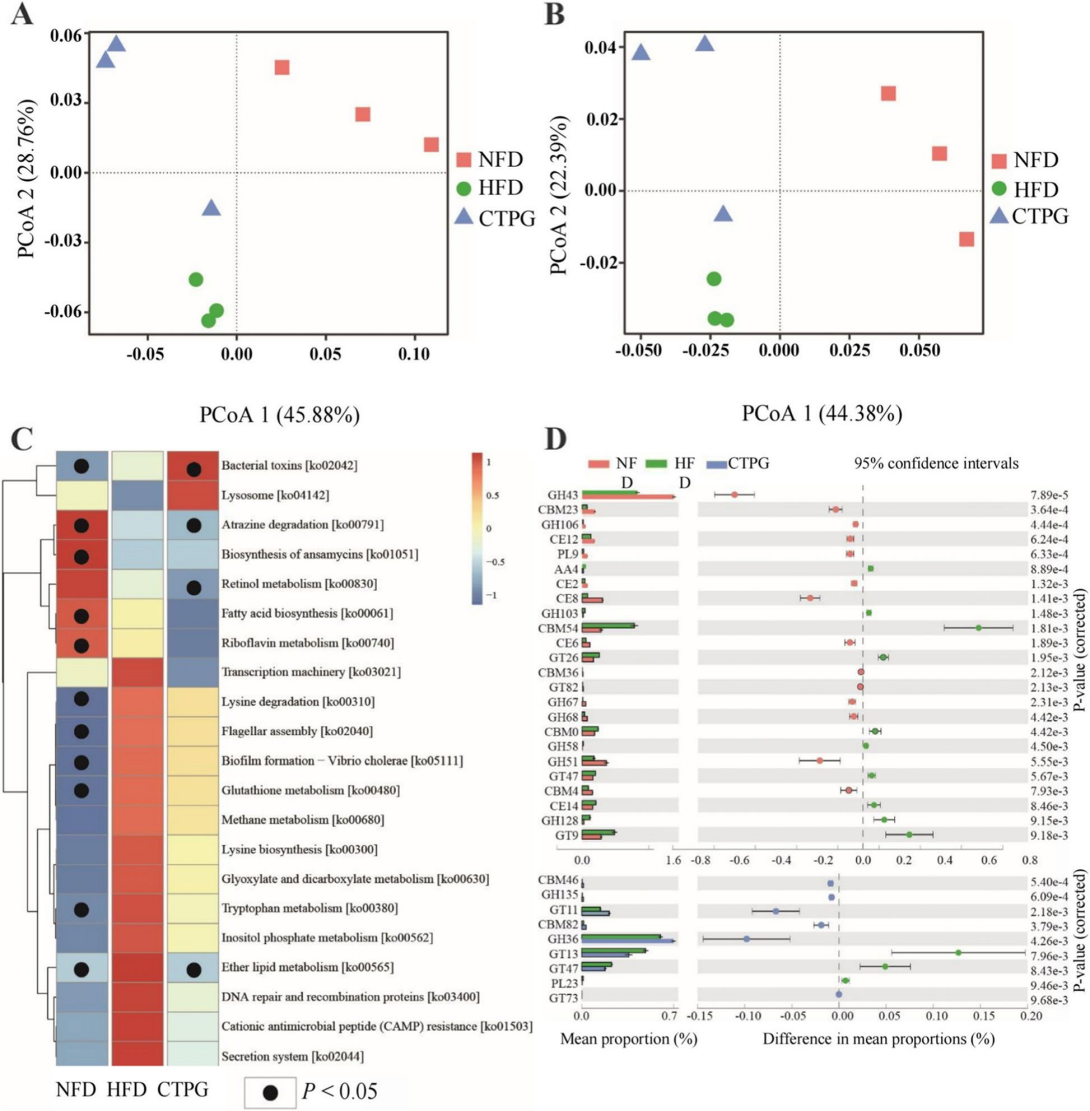
CTPG regulates gut microbial function of HFD mice. **A** PCoA analysis at KEGG orthologs level. **B** PCoA analysis in CAZy families. **C** Heatmap of the relative abundances of the metabolic pathways with the criteria of LDA > 2. The black dots mean significant difference (*p* < 0.05) between HFD and NFD groups or between HFD and CTPG groups. (D) Significant difference analysis in CAZy families

The gut microbiota structure changes are always accompanied with the alternation of gut microbial function. Both Bray–Curtis PCoA based on Kyoto Encyclopedia of Genes and Genomes (KEGG) orthologs group (KOs) and Carbohydrate-Active enZYmes (CAZy) families showed that the clusters of metabolic pathways were clearly separated among NFD, HFD, and CTPG groups (Fig. 8A&B).

The linear discriminant analysis (LDA) Effect Size (LEfSe) was used to analyze the metabolism on gut microbial function, and 21 metabolic pathways were screened with the criteria of LDA > 2 (Fig. 8C). Compared to NFD group, 14 metabolic pathways including transcription machinery, lysine degradation, flagellar assembly, biofilm formation-Vibrio cholera, glutathione metabolism, methane metabolism, lysine biosynthesis, glyoxylate and dicarboxylate metabolism, tryptophan metabolism, inositol phosphate metabolism, ether lipid metabolism, DNA repair and recombination proteins, cationic antimicrobial peptide (CAMP) resistance and secretion system were increased in gut of HFD mice, in which six metabolic pathways were significantly increased (*p* < 0.05). After CTPG supplementation, the 14 metabolic pathways were reversed at different degree, in which ether lipid metabolism pathway was recovered to normal degree (Fig. 8C). Moreover, CAZy database annotation results showed that 24 CAZy families were significantly changed in gut of HFD mice compared with NFD mice, in which 9 CAZy families were changed by CTPG supplementation (*p* < 0.05) (Fig. 8D). These results indicated that CTPG supplementation could partly recover the functions of the gut microbiome in HFD mice.

**Fig. 9:**
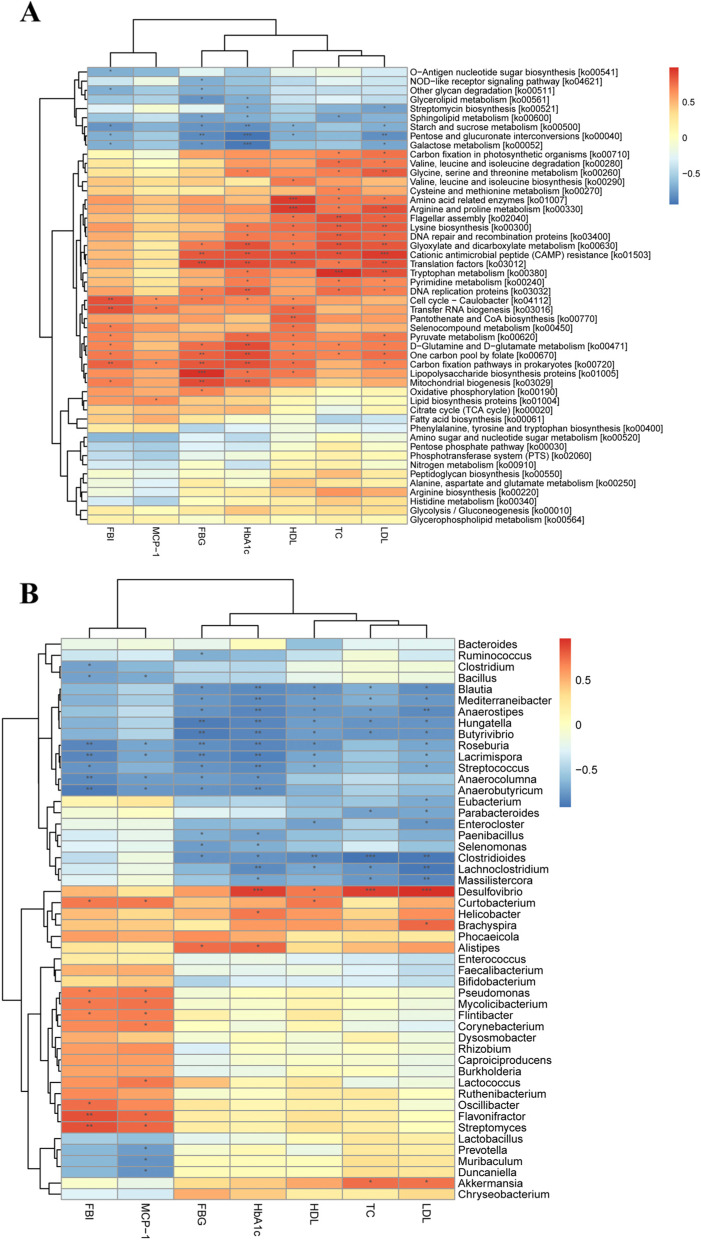
Functional correlation analysis of metagenomics COG and KEGG with CTPG treatment. **A** Correlation analysis was conducted between the top 50 bacteria with high abundance and MCP-1, FBI, FBG, HbA1c, HDL-C, TC and LDL-C. **B** Correlation analysis between COG and KEGG function and MCP-1, FBI, FBG, HbA1c, HDL-C, TC and LDL-C (red, positive correlation; blue, negative correlation). ^*^p < 0.05, ^**^p < 0.01 and.^***^p < 0.001

In addition, spearman correlation cluster analysis was conducted for OUT data, and correlation analysis was conducted between the top 50 bacteria with high abundance and MCP-1, FBI, FBG, HbA1c, HDL-C, TC and LDL-C (Fig. 9A). Correlation analysis showed that *Alistipes*, *Helicobacter*, *Desulfovibrio*, *Curtobacterium*, *Brachyspira*, *Akkermansia* were significantly positively correlated with FBG, HbA1c, HDL-C, TC and LDL-C. *Curtobacterium*, *Pseudomonas*, *Mycolicibacterium*, *Flintibacter*, *Flavonifractor* and *Streptomyces* were significantly positively correlated with MCP-1 and FBI. However, *Blautia*, *Anaerostipes*, *Mediterraneibacter*, *Hungatella*, *Butyrivibrio*, *Roseburia*, *Lacrimispora*, *Streptococcus*, *Parabacteroides*, *Clostridioides*, *Lachnoclostridium* and *Massilistercora* were significantly negatively correlated with FBG, HbA1c, HDL-C, TC and LDL-C, and *Bacillus*, *Roseburia*, *Lacrimispora*, *Streptococcus*, *Anaerocolumna* were significantly negatively correlated with MCP-1 and FBI.

**Fig. 10:**
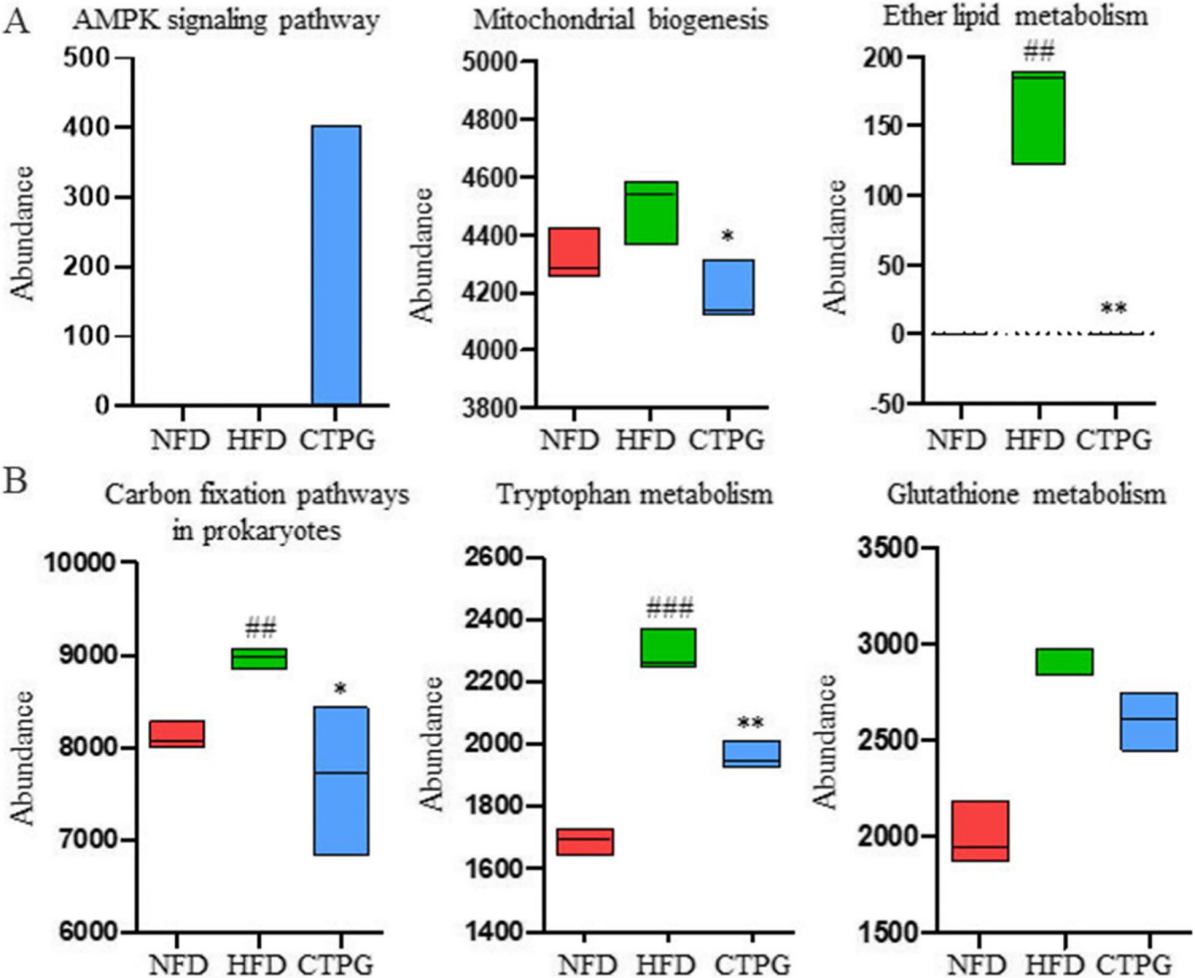
CTPG regulated the metabolic pathways including glycolipid metabolism, inflammation, and short-chain fatty acids through gut microbiota. **A** AMPK signaling pathway, mitochondrial biogenesis, ether lipid metabolism. **B** Carbon fixation pathways in prokaryotes, tryptophan metabolism, glutathione metabolism. Data are expressed as mean ± SD. ^#^*p* < 0.05, ^##^*p* < 0.01 and ^###^*p* < 0.001, HFD group versus NFD group. ^*^p < 0.05, ^**^p < 0.01 and ^***^p < 0.001, CTPG and MET groups versus HFD group

In KEGG pathway analysis, gut microbial function was reversed after CTPG supplementation, the lysine biosynthesis, glyoxylate and dicarboxylate metabolism, tryptophan metabolism, lipopolysaccharide biosynthesis proteins, mitochondrial biogenesis were positively correlated with glucose and lipid metabolism. Proteins enriched in AMPK signaling pathway, mitochondrial biogenesis and ether lipid metabolism were also positively correlated with MCP-1 and FBI (Fig. 9B, 10A). Similarly, we found that SCFAs associated carbon fixation pathways in prokaryotes, tryptophan metabolism and glutathione metabolism were down-regulated in HFD mice treated with CTPG (Fig. 10B). The results further indicated that CTPG improved glucose, lipid metabolism, inflammation and insulin sensitivity in HFD mice by regulating key gut microbiota.

## Discussion

Obesity is characterized by increased adipose tissue mass and is often accompanied by a number of other metabolic disorders [4, 5, 56]. Currently, there are serious side effects induced by anti-obesity drugs. Therefore, it is necessary to develop safe and efficient anti-obesity drugs with low side effects. Natural products have become the main resource and strategy for screening anti-obesity drugs. In present study, to evaluate the effects of CTPG as potential drugs to prevent obesity, we focused on the regulation of obesity and insulin resistance in vitro and in vivo.

Many studies have shown that the mechanisms of anti-obesity are involved in inhibiting the proliferation and differentiation of preadipocytes [57–59]. Similarly, our results showed that CTPG significantly inhibited the differentiation of 3T3-L1 preadipocytes into adipocytes, and decreased lipid droplet accumulation, triglyceride and NEFA levels in 3T3-L1 adipocytes. Moreover, CTPG also increased glucose consumption and decreased NEFA levels in IR-3T3-L1 adipocytes.

Adipogenesis is a process that preadipocytes differentiate into mature adipocytes through the regulation of multiple transcription factors and lipogenic genes. C/EBPα is one of the CEBP members and expressed at the early stage of adipogenesis [60].At the late stage of differentiation, downstream key enzymes controlling fatty acids synthesis including FAS and ACC are expressed to promote adipogenesis and lipid accumulation in adipocytes [61, 62]. Pref-1 is an early negative regulator of adipogenic differentiation [63]. In this study, the expression of C/EBPα, ACC and FASN in 3T3-L1 mature adipocytes was inhibited and the expression of Pref-1 was increased by CTPG, suggesting that lipid accumulation and adipogenic differentiation were suppressed. In addition, the expression of HSL was greatly increased by CTPG, which is a key regulatory enzyme in controlling lipolysis in adipose tissue [64]. These results indicated that CTPG had strong effects on anti-lipogenesis and improving insulin sensitivity through inhibiting the synthesis of triglycerides and the release of NEFA by down-regulating adipogenesis or accelerating lipolysis.

The expansion of adipose depots can be driven either by hypertrophy or hyperplasia in obesity [65]. We further studied the effects of CTPG in obese mice induced by HFD. CTPG treatment not only reduced fat mass and size of adipose tissue and hepatic steatosis in obese mice, but also effectively improved blood glucose, lipid and insulin tolerance levels, suggesting that CTPG improved obesity, glucolipid metabolism and IR in obese mice. It has demonstrated that obese mice have higher rates of lipogenesis and cholesterol synthesis, which resulted in the increased serum cholesterol levels [66]. Consistently, TC levels in HFD mice were significantly down-regulated and AMPKαactivity was activated after CTPG treatment in our study. This is consistent with the previous reported results that AMPKα inhibits TC synthesis [67–70]. AMPK activation suppresses several lipogenic pathways, leads to the activation of fatty acid oxidation, and improves insulin sensitivity and metabolic health in liver and adipose tissue [69, 71, 72]. We also found CTPG improved glucose and lipid metabolism in HFD mice by AMPK signaling pathway, fatty acid biosynthesis and mitochondrial biogenesis through metagenomics data in KEGG functional annotation. Although the precise mechanisms of action remain to be elucidated, given the present results, the increase in whole body fatty acid oxidation might account for, at least in part, the reduction in fat accumulation by CTPG through an activation of AMPKα in the liver.

Recent studies strongly support that adipose tissues dysfunctions secreting high levels of free fatty acids (NEFA) and inflammation adipokines under HFD or obese conditions are crucial players in lipotoxicity, glucose metabolism disorder and insulin resistance [73]. MCP-1 as one of the crucial adipocytokines, accelerates macrophage and T lymphocytes infiltration into the sites of inflammation in adipose tissue [74]. Here, we found that CTPG decreased the MCP-1 levels in 3T3-L1 adipocytes and eWAT, which was positively correlated with lipid biosynthesis pathway.

Furthermore, adipose tissue inflammation is also involved in the regulation of the insulin pathway to affect systemic glucose tolerance and insulin sensitivity [19, 75]. Insulin-stimulated glucose uptake in the adipocyte is critically dependent on the IRS1/Akt axis [17]. The imbalance of insulin signaling pathway caused the impaired translocation of GLUT4, which resulted in IR and T2DM [19, 76]. Our results showed that CTPG stimulated IRS1/Akt insulin signaling pathway in WAT, which subsequently promoted the translocation of GLUT4 to membrane and improved IR and glucose homeostasis in HFD mice.

Changes in the gut microbial composition, structure and function are associated with increased susceptibility to obesity [77, 78]. The majority of the gut microbiota consists of five phyla including *Bacteroidetes*, *Firmicutes*, *Actinobacteria*, *Proteobacteria*, and *Verrucomicrobia* [79], which play a pivotal role in regulating metabolic processes [80]. In this study, HFD obviously increased the relative abundance of *Proteobacteria* and *Actinobacteria* phyla, while CTPG supplementation decreased the abundance of *Proteobacteria* phylum in HFD mice.

Increasing evidence has linked glucose, lipid homeostasis and insulin sensitivity to the gut microbiota composition, which may play an important role in the pathogenesis of metabolic diseases, such as obesity, IR [77, 81, 82]. Correlation analysis showed that *Roseburia* and *Lacrimispora* are the two genera most closely related to glucose, lipid metabolism and IR, and they might play an important role in the CTPG-mediated improvement of glucose, lipid homeostasis and insulin sensitivity. Studies showed that *Roseburia* was significantly correlated with body fat percentage and HbA1c [83]. In our study, *Roseburia* was also negatively correlated with HbA1c, LDL-C and FBI. It has been reported that *Blautia*, *Anaerostipes* and *Roseburia* were negatively correlated with fasting blood glucose and insulin resistance index levels [84], and this is consistent with our results, which suggest that CTPG might improve glucolipid metabolism and insulin sensitivity via stimulation of IRS1/Akt insulin signaling pathway and modulation of gut microbiota. In the present study, *Roseburia* and *Lacrimispora* were significantly negatively correlated with MCP-1. Additionally, the CTPG supplementation significantly increased the abundances of *Roseburia* and *Lacrimispora*, which were similar with NFD groups. *Roseburia* reported as the anti-obesity and anti-inflammatory bacteria in mice [85]. This suggests that *Roseburia* might play an important role in the CTPG-mediated anti-inflammatory effects. This is consistent with KEGG analysis of gut microbiota. However, the mechanism remains to be further explored. These findings suggested that CTPG supplementation not only changed the gut microbial structure but also function of gut microbiota in obese mice.

## Conclusions

In conclusion, we demonstrated that supplementation with CTPG significantly suppressed adipocyte differentiation, adipogenesis and inflammation in 3T3-L1 adipocytes, as well as glucose consumption, lipid metabolism and insulin resistance in IR-3T3-L1 cells. Furthermore, CTPG ameliorated HFD-induced obesity by inhibiting hypertrophy and hyperplasia of adipose tissue, inflammation and improving glucolipid metabolism and insulin sensitivity, changing structure and function of gut microbiota in obese C57BL/6 N mice. Our results suggested that CTPG might be a potential candidate for the treatment of obesity and IR.

## Supplementary Information

**Additional file 1.**

**Additional file 2.**

## Data Availability

All data generated for this study are included in this published article and its supplementary information files. The datasets presented in this study can be found in online repositories. The names of the repository/repositories and accession numbers can be found below: NIH National Library of Medicine and BioProject; PRJNA773205, SAMN22456120, SAMN22456119, SAMN22456116, SAMN22456113, SAMN22456118, SAMN22456114, SAMN22456115, SAMN22456117 and SAMN22456112.

## Abbreviations

DM: Diabetes mellitus
T2DM: Type 2 diabetes mellitus
IR: Insulin resistance
HFD: High-fat diet
NFD: Normal-fat diet
MET: Metformin
CTPG: Phenylethanoid glycosides from *Cistanche tubulosa*
MTT: Methyl thiazolyl tetrazolium
FBS: Fetal bovine serum
NBS: New born calf serum
PBS: Phosphate buffer sodium
IBMX: 3-Isobutyl-1-methyl-7H-xanthine
DEX: Dexamethasone
RG: Rosiglitazone
C/EBPα: CCAAT enhancer-binding protein alpha
ACC: Acetyl-CoA carboxylase
FASN: Fatty acid synthase
Pref-1: Preadipocyte factor-1
HSL: Hormone sensitive lipase
H&E: Haematoxylin & eosin
ELISA: Enzyme-linked immunosorbent assay
WAT: White adipose tissue
eWAT: Epididymal
pWAT: perirenal WAT
iWAT: Inguinal WAT
BAT Brown: adipose tissue
OGTT: Oral glucose tolerance test
ITT: Insulin tolerance test
QUICKI: Quantitative insulin sensitivity check index
HOMA2: Homeostasis model assessment-2
OD: Optical density
TG: Triglyceride
TC: Total cholesterol
NEFA: Nonesterified fatty acid
AST: Aspartate aminotransferase
ALT: Alanine aminotransferase
GHbA1c: Glycated hemoglobin A1c
IRS1: Insulin receptor substrate 1
Akt: Protein kinase B
GLUT: Glucose transporter
AMPK: AMP-activated protein kinase
MCP-1: Monocyte chemoattractant protein-1
SCFAs: Short chain fatty acids
CAZy: Carbohydrate-Active enzymes
KEGG: Kyoto Encyclopedia of Genes and Genomes
