# Supplementary material for: Cistanche tubulosa phenylethanoid glycosides suppressed adipogenesis in 3T3-L1 adipocytes and improved obesity and insulin resistance in high-fat diet induced obese mice
Source: BMC Complement Med Ther. 2022 Oct 13;22:270. doi: 10.1186/s12906-022-03743-6 (PMC9564091; doi:10.1186/s12906-022-03743-6)
Supplement: Supplementary file 1 — Additional file 1. [file 12906_2022_3743_MOESM1_ESM.docx]

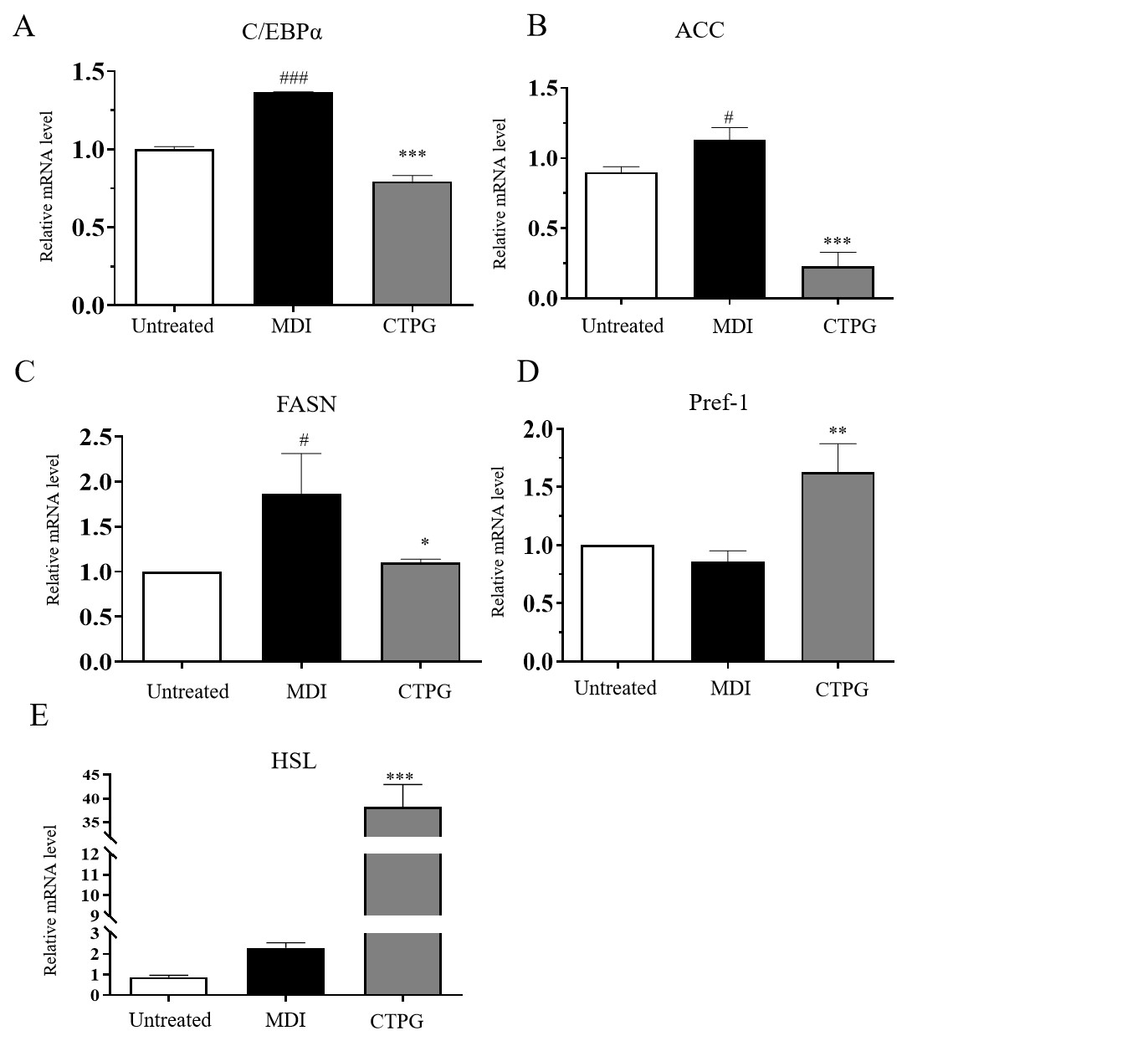


**Figure S1.** Effect of CTPG on the expression levels of lipogenesis genes in 3T3-L1 adipocytes. 3T3-L1 cells were treated with 100 μg/ml CTPG for 72h and the mRNA levels for *C/EBPα* (A) , ACC (B), FASN (C), Pref-1 (D) and HSL(E) were analyzed by qRT-PCR*.*

**Table S1.** The gene-specific primers.

| **Gene** | **Primer sequences (5' to 3' )** | |
| --- | --- | --- |
| *β-actin* | F:GGCTGTATTCCCCTCCATCG | R:CCAGTTGGTAACAATGCCATGT |
| *C/EBPα* | F:CAAGAACAGCAACGAGTACCG | R:GTCACTGGTCAACTCCAGCAC |
| *ACC* | F:GCCTCAGGAGGATTTGCTGT | R:AGGATCTACCCAGGCCACAT |
| *FASN* | F:GGAAGTTGCCCGAGTCAGAG | R:CTTTCCAGACCGCTTGGGTA |
| *HSL* | F:TCAATGGAGACACTTGGCCC | R:TTGGCTTCAGCCTCTTCCTG |
| *Pref-1* | F:AGTGCGAAACCTGGGTGTC | R:GCCTCCTTGTTGAAAGTGGTCA |
