# Supplementary material for: Cistanche tubulosa phenylethanoid glycosides suppressed adipogenesis in 3T3-L1 adipocytes and improved obesity and insulin resistance in high-fat diet induced obese mice
Source: BMC Complement Med Ther. 2022 Oct 13;22:270. doi: 10.1186/s12906-022-03743-6 (PMC9564091; doi:10.1186/s12906-022-03743-6)

Full-length blots for Figure 4H

Total protein was isolated from livers and AMPKα protein expression and phosphorylation of AMPKα were detected by Western blot. GAPDH was used as control. The other three bands without number are HFD mice treated with other drugs.

GAPDH
(1 represents NFD; 2 represents HFD; 3 represents MET, 4 represents 300 mg/kg CTPG.)

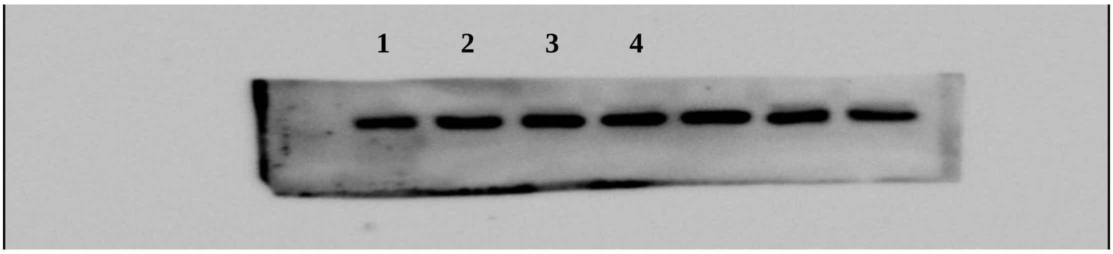

AMPKα
(1 represents NFD; 2 represents HFD; 3 represents MET, 4 represents 300 mg/kg CTPG.)

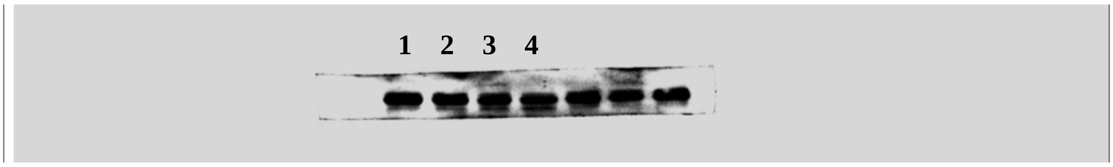

pAMPKα
(1 represents NFD; 2 represents HFD; 3 represents MET, 4 represents 300 mg/kg CTPG.)

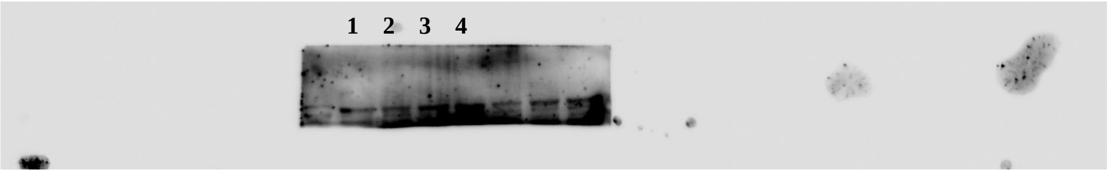

Full-length blots for Figure 5I

Total protein was isolated from eWAT to detect protein expression and their phosphorylation in insulin signaling pathway by Western blot. GAPDH was used as control. The other three bands without number are HFD mice treated with other drugs.

GAPDH
(1 represents NFD; 2 represents HFD; 3 represents MET, 4 represents 300 mg/kg CTPG)

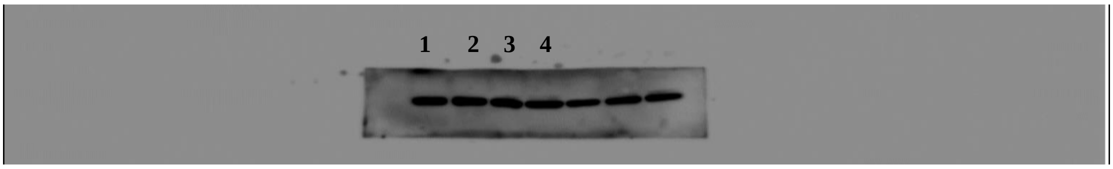

IRS1

(1 represents NFD; 2 represents HFD; 3 represents MET, 4 represents 300 mg/kg CTPG)

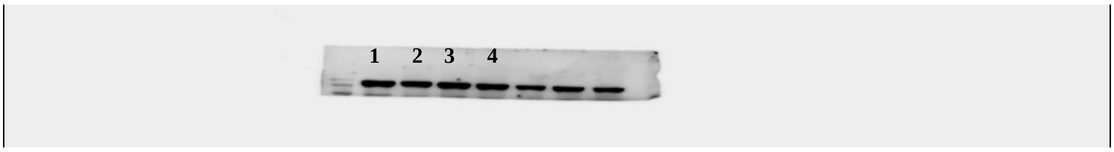

pIRS1

(1 represents NFD; 2 represents HFD; 3 represents MET, 4 represents 300 mg/kg CTPG)

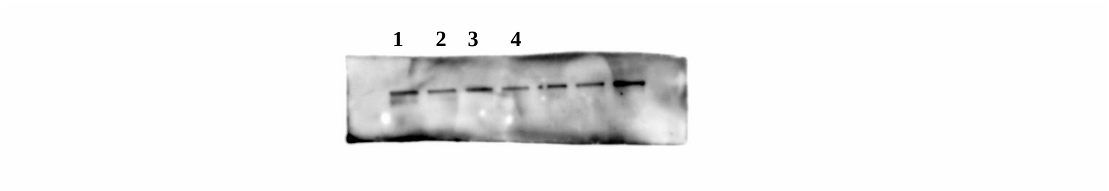

GLUT4

(1 represents NFD; 2 represents HFD; 3 represents MET, 4 represents 300 mg/kg CTPG)

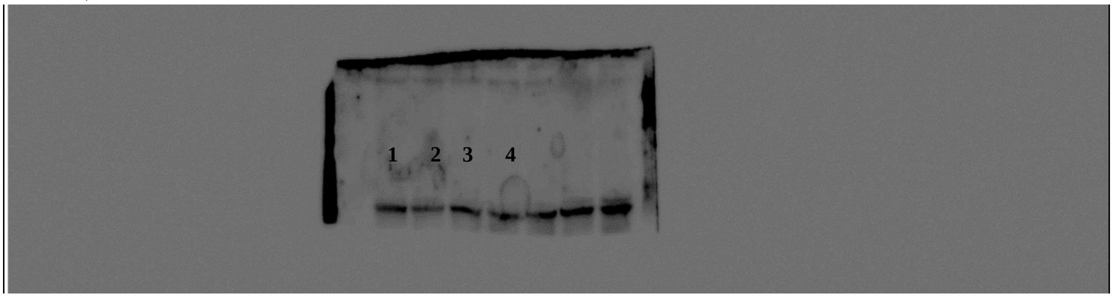

Akt

(1 represents NFD; 2 represents HFD; 3 represents MET, 4 represents 300 mg/kg CTPG)

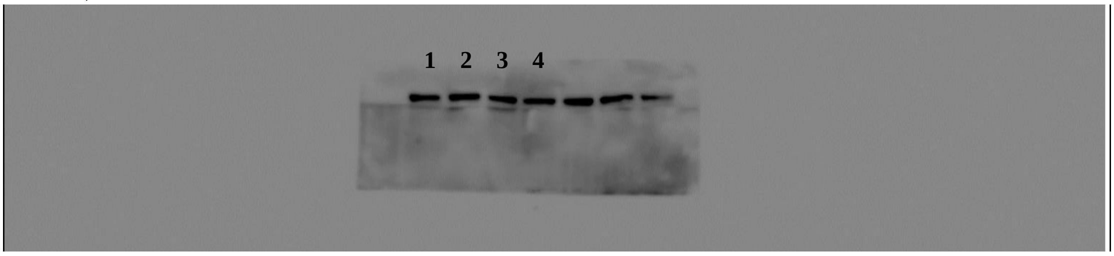

pAkt (Ser473)

(1 represents NFD; 2 represents HFD; 3 represents MET, 4 represents 300 mg/kg CTPG)

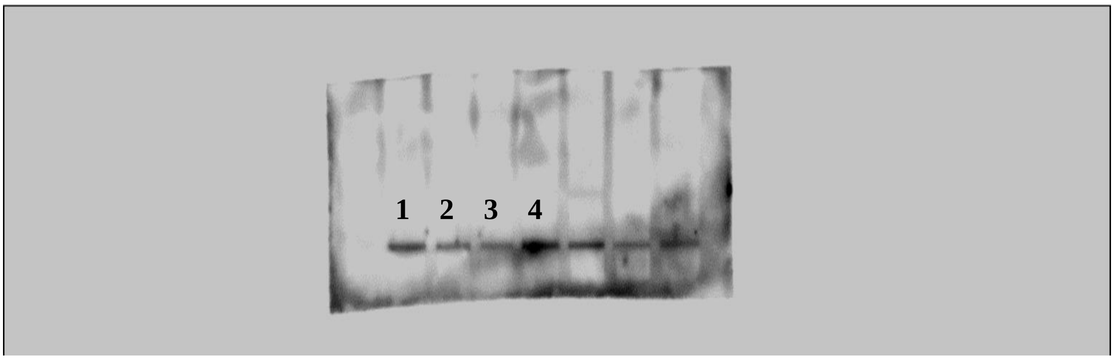

pAkt (Thr308)

(1 represents NFD; 2 represents HFD; 3 represents MET, 4 represents 300 mg/kg CTPG)

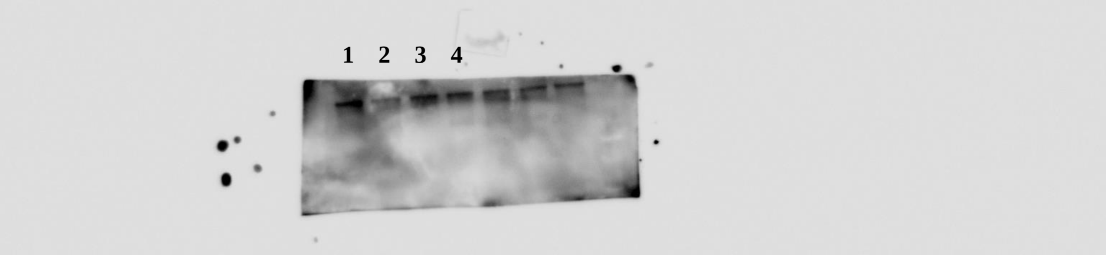

Supplement: Supplementary file 2 — Additional file 2. [file 12906_2022_3743_MOESM2_ESM.pdf]
